# Annotations

**Published:** 1911-07-01

**Authors:** 


### ANNOTATIONS.

### The Medical Council and the Public Schools.

Last month the General Medical Council accepted by a majority of twenty-four votes to five the (modified) motion which was proposed by Sir Henry Morris. The amended motion thus passed reads as follows: "That it be an instruction to the Students' Registration Committee that when a secondary school has been inspected and recognised by a licensing body, and has applied to be placed on the list of approved institutions in which medical study may be commenced, the Registration Committee shall consider every such application, and, when satisfied that the education in such secondary school is of a sufficient standard, shall recommend such secondary school to be placed by the Council on the list of approved institutions." Thus concludes definitely and decisively a state of things which has long been in need of alteration. The Medical Council, in thus accepting a very necessary reform of procedure, has adopted the only course consistent with its own dignity, and one which has the approval and energetic support of the most progressive of our Public School headmasters. The Council, in thus "recognising" in a technical sense the schools to which this resolution refers, is merely recognising an accomplished fact, and one to which it would have been folly to remain officially blind.

### "An Imperial Dispensatory."

We understand that in a short time the new British Pharmaceutical Codex will be ready for publication. It is four years since the experimental first issue of this work obtained a marked success, and it is now likely to be very widely recognised as an "Imperial Dispensatory." Based on the pharmacopœias of Great Britain and the U.S.A., the new Codex contains specially prepared descriptions of all drugs and medicines in common use, with chemical and dispensing notes and an extensive formulary of galenical preparations which are not official in the two pharmacopœias. It is worthy of note that all the B.P.C. preparations have now been made and thoroughly tested before inclusion in the work. An entirely novel feature of the new codex is a pharmacological and therapeutic index, which provides a useful classification of remedies, all the medicaments being grouped in one alphabetical list in accordance with their actions and uses. Both in hospital and private dispensaries the work is one of great usefulness to prescriber and dispenser alike, and the new edition is likely to become indispensable to the dispenser's library.

### The Origin of Species.

Physicists no longer hold to the Daltonian atomic theory which reigned supreme a dozen years ago; and pure Darwinian evolutionists are likewise becoming less dogmatic and less numerous. The axiom that *Natura non facit per saltum* is receiving some severe shocks from the researches of modern philosophers. In India, Brevet-Colonel Firth, of the Royal Army Medical Corps, has been conducting experiments and collecting data as to the origin of varieties and species among certain flowering plants. He believes, as do many of the new school of thinkers, that new species may and do arrive by hybridisations and by mutations; and that the Darwinian conception of extremely small variations accumulated and perpetuated by natural selection is one which requires a longer period of time than can probably be assigned to the existence of living forms upon the globe. In the majority of cases he thinks hybridisation accounts for new species; yet the mutation conception, undeveloped and imperfectly understood though it still is, co-ordinates a mass of hitherto unintelligble facts. Admitting the existence of mutants, which we must, we recognise that the greater number perish, because they do not meet with all the conditions necessary for their survival. In other words, there is a natural selection among species by which a few aberrants are selected for survival; those not selected perish. Colonel Firth looks forward to a not far distant day when man will be able to help, if not indeed to supplant, nature in the evolution of organisms.

### Spheres and Syringes.

The enterprise of the business managers of modern newspapers is by now as familiar as it is (frequently) ingenious; and that of surgical instrument makers, if less conspicuous to the public, is at least well-known to the profession. The combination of illustrated journalism with the surgical instrument business is, however, a distinct novelty. If it succeeds, presumably, it will be considered to have justified itself; but it is certainly difficult to trace any inevitable connection between weekly newspapers and hypodermic syringes, or to see why those who want one should necessarily want to buy the other at the same time and through the same agency. Whether the syringe is to be regarded as given away with the paper, or the paper with the syringe, one cannot presume to judge. The offer that has been made in circulars distributed to members of the profession is to supply a new pattern of hypodermic syringe, which is claimed to be an improvement on all existing syringes, and to be worth half-a-guinea, together with a year's issue of the paper in question, for a sum greater by two shillings and twopence than the subscription rate for the paper alone. It is held out as an inducement that the syringe can for the present be obtained in no other way, as the makers are not supplying it except through the proprietors of the newspaper. To this singular proposal no objection whatever can be taken; it is open to everyone to cry his wares, whether they be illustrated papers or hypodermic syringes or anything else, at such terms as he deems he can get. The public, in its turn, has the right to buy what it likes. The syringe is quite undescribed in the advertisement, and there is no means of judging whether its value is more or less than half-a-guinea, or than two-and-twopence. Probably, however, it is fairly safe to restrict the number of orders which can be executed on these terms to five thousand; the offer is limited to that number of applicants.

